# The Potential of Intraepithelial Lymphocytes as Immunological Targets for the Induction of Innate and Adaptive Memory Responses Through Mucosal Vaccination and Prime Boost

**DOI:** 10.3390/vaccines14070579

**Published:** 2026-06-30

**Authors:** Gloria G. Guerrero-Manríquez, Marco A. De León-Nava

**Affiliations:** 1 Unidad Académica de Ciencias Biológicas, Universidad Autónoma de Zacatecas, Zacatecas 98066, Zacatecas, Mexico; 2 Departamento de Innovación Biomédica, Centro de Investigación Científica y de Educación Superior de Ensenada (CICESE), Baja California, Mexico

**Keywords:** mucosal immunity, epithelial tissues, intraepithelial lymphocytes (T-IELs), adaptive immunity, mucosal vaccination, prime boost protocols

## Abstract

The mucosal surfaces of the different tracts of the human body occupy a vast interface (around 400 m^2^), and defense at these sites represents the first line of immune defense. It is at the interface of these surfaces that a cellular and molecular crosstalk among different players is accomplished: the microbiota, the mucosal epithelial, and the immune system. Different lymphocyte populations are present on these surfaces as sentinels, ready to act upon any insult that threatens body homeostasis. One of them are the T-cell-derived intraepithelial lymphocytes (T-IELs), gatekeepers at the mucosal epithelium of the common mucosal system (MALT) (gut, lung, and urogenital tract). These lymphocytes are divided into natural, non-conventional (CD4+/CD8+, TCR αβ, γδ, αα) and induced T-IELs or conventional (CD4+/CD8+ TCR αβ). The most remarkable and distinguishing properties of these innate lymphocyte populations are a triad of attributes: innate, cytotoxic, and memory-protective immune responses. T-IELs are a disregarded population of innate cells, ready to act, for rapid microbial clearance, and an effective support for any mucosal invader. Despite this, T-IELs are an underutilized immunological target that needs further in-depth investigation into their role in inducing fast, rapid clearance of mucosal pathogens, and protective and immune memory (innate and adaptive). The present commentary aims to put into context this emerging potential of T-IELs as immunological targets.

## 1. Introduction

**Figure 1:**
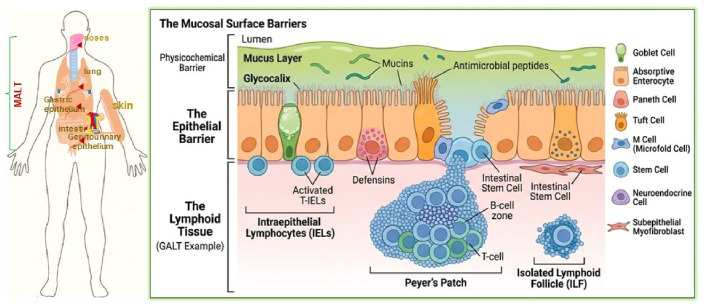
**The vast mucosal surface of 400 mt^2^ of the mucosa-associated lymphoid tissue (MALT),** comprising the physicochemical barrier (mucins, mucous, and glycol calix), epithelial tissue, in which exist different populations of cells including epithelial, goblet, Paneth, tuft, M, myofibroblast, neuroendocrine and stem cells, and lymphoid tissue, populated with activated T-cell-derived intraepithelial lymphocytes (T-IELs). These T-IELs are specialized sentinels, gatekeepers, ready to act to immune modulate against a myriad of dietary antigens and mucosal invaders (inset). AI Gemini 3.1. Pro Advanced.

Humans are constantly exposed to an environment containing countless microbial organisms, including non-pathogenic commensal microbiota and a wide variety of infectious agents [1,2,3,4,5]. Contact with these typically occurs on body surfaces, both external and internal, such as the skin and mucous membranes of the intestinal, respiratory, and genitourinary tracts; these mucosal surfaces occupy a vast interface (around 400 m^2^) [1,2] (Figure 1). Due to this contact, the immune system has developed specialized characteristics for protection against potential invaders, generating dynamic interactions between the host and microorganisms [4,5,6]. Many properties of the adaptive immune system, such as organized lymphoid structures, are believed to have begun their evolution in the vertebrate gut in response to its interaction with the microbiota [7,8,9]. Therefore, the immune defense is vital to maintain homeostasis and tolerance at these compartments in which the immune system, armed with innate lymphocytes, establishes a molecular and cellular crosstalk for the induction of protective and immunological memory [1,2,3,4,5] (Figure 1). How is this immune response accomplished at these compartments? In general, the immune response initiates at the level of the physicochemical barriers (mucus and cilia) and is accomplished by innate cell alveolar macrophages, epithelial cells, and antimicrobial peptides, which detect pathogens, releasing inflammatory and danger signals for recruiting more innate immune cells (i.e., neutrophils) to the site of infection [7,8,9,10]. Dendritic cells also play a very important role as APCs for the induction of a pro-inflammatory response and for the activation of lymphocytes (T and B cells), NK cells, and the different subsets of T cells, those that migrate from the periphery or from the gut such as Th17 cells and IL-17 inducers, as a type of adaptive immune response [2,6]. At the level of the MALT, the induction of a mucosal immune response, for example, in the lung, is led by alveolar macrophages and dendritic cells, as antigen-presenting cells. The pulmonary innate immune cells (ILCs) and neutrophils participate in the induction of an inflammatory response upon infection (microbial or viral) through the secretion of several cytokines (IL-1β and IL-23), to activate γδ-IL-17 T cells and IL-17 [11,12,13,14,15,16], chemokines, and the expression of integrins, for the extravasation of leucocytes to the site of infection. Alveolar macrophages, once activated, produce IL-12, whereas NK cells produce interferon gamma, and other innate lymphocyte populations (IC2 and IC3) also secrete cytokine Th1 or Th2 types that participate in the differentiation of naïve CD4^+^ T cells [16,17,18,19]. On the other hand, in close relationship with the above key players of the innate immune response in the different compartments of the upper airways, the pulmonary microbiota also plays a pivotal role in shaping and regulating the pulmonary innate and adaptive immune response [16,17,18,19,20,21] (Figure 1).

In gut/GALT (gut-associated lymphoid tissue), the immune response initiates with the molecular recognition of pathogen-associated molecular patterns (PAMPs) on a myriad of microbial/viral antigens, and the pattern of repeated receptors (PRRs) on the APCs (DCs, macrophages, and epithelial cells) [22,23,24]. Thereafter, the sampled antigen is transported to Peyer’s patches, to activate naïve CD4^+^ T and B cell lymphocytes, followed by the activation of NK, iNKT and CD8^+^ T cells, leading to signaling downstream, NF-kB translocation, and a pro-inflammatory induction, represented by IL-6, TNF-α, IL-12, IL-4, for naive CD4^+^ Th differentiation [1,2,3,9,10,25,26] and further effector immune response, including secretory IgA and APCs inducing cytokines (IL-12, IFN-γ, TGF-β, TNF-α, IL-6, and IL-4) for the differentiation of T and B lymphocyte populations toward Th1, Th2, Th3, and Th17 [1,2,3,4,13,14,20,21,22,23,24]. Moreover, in GALT there is a specialized lymphocyte population, the intraepithelial lymphocytes (T-IELs) [24,25,26,27,28], characterized by cytotoxic T cell-related properties, NK cell receptors, high expression of integrins, and homing receptors [24,25,26,27,28,29]. Among the repertoire of lymphocytes in the mucosal epithelium, playing a key role in homeostasis, tolerance and regulatory immunity at the mucosal epithelium, are the T-cell-derived intraepithelial lymphocytes (T-IELs), the functionality of which depends of the interaction with microflora and epithelial cells, thus providing a pool of resident T-IELs with a threshold of activation against a myriad of antigens threatening the mucosal epithelium of the lung, gut and reproductive tracts [13,20,23,27,28]. The specialized crosstalk of T-IELs at the interface of these surfaces can be important for the search and design of immunotargets [24,26,30,31,32,33]. Mucosal vaccination (oral/nasal) of the candidate’s vaccine, along with the prime boost, could facilitate the immunological targeting of T-IELs and potentiate their innate, effector properties (cytotoxic properties), a hallmark of T-IELs which remains unexplored and underutilized [20,22,25,28,32,33,34,35,36] (Figure 1).

## 2. Heterogeneity of Intraepithelial Lymphocytes (T-IELs) in MALT

**Figure 2:**
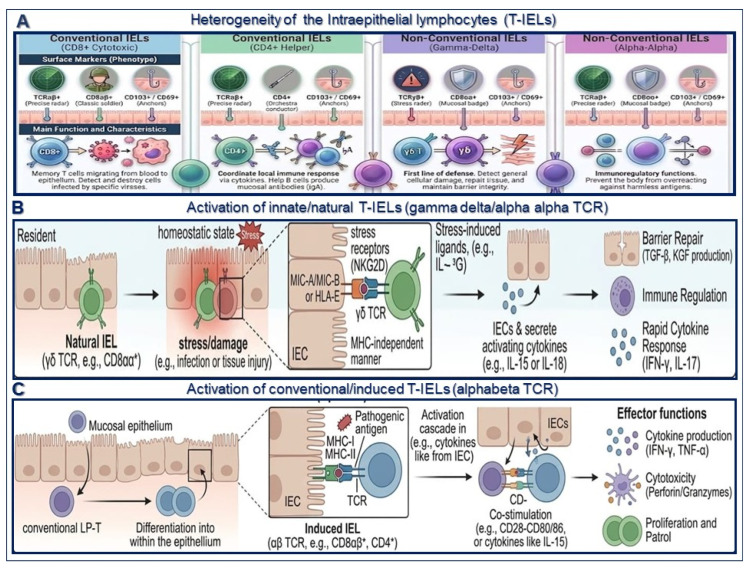
**Heterogeneity of T-cell-derived intraepithelial lymphocytes (T-IELs) in MALT.** The proportions of T-IELs either in bronchus-associated lymphoid tissue (BALT) or in gut-associated lymphoid tissue (GALT) are similar (20 IELs/100 epithelial cells approximately). There are two types of T-cell-derived IELs, natural and induced T-IELs (**A**). Natural T-IELs express TCRγδ or TCRαβ but do not express either CD4+ or CD8αβ+. In addition, these IELs express homodimers of CD8αα and CD8+ cytotoxic/suppressor T cells, and usually predominate over CD4+ T-helper cells, but this predominance is less marked in bronchial epithelium than in the intestine. The induced IELs are derived from circulating conventional CD4+ or CD8+ T cells, and with the help of gut-homing receptors, including αEβ7 integrin and CC-chemokine Receptor 9 (CCR9) and their ligand, which are produced by IECs, the mature induced IEL precursors finally migrate to the intestinal epithelium after the post-thymic differentiation process. Double-positive T-IELs (CD4^+^CD8αα IELs or DP IELs), which are a kind of CD4^+^T-IELs with some functions of CD8^+^IELs, are restricted through major histocompatibility complex (MHC) class II and class I, respectively. Moreover, in the intestine, a substantial amount of T-IEL expresses γδ T-cell receptors, while almost all human bronchial T-IELs express αβγδ T-cell receptors, and are also equipped with several properties, including innate and cytotoxic capacity, and high threshold activation. Mechanism of activation of the natural T-IELs (Innate-like) IELs. These cells (such as TCRγδ+ or TCRαβ+CD8αα+) migrate to the epithelium directly from the thymus without prior antigen exposure in the periphery, and are independent of MHC presentation. They act as innate-like first responders, for example to stress, or tissue injury, tissue damage. Many T-IELs express the NKG2D receptor, which functions as a powerful co-stimulatory or direct activation pathway. When epithelial cells are infected or stressed, they upregulate stress ligands (such as MICA/MICB). NKG2D binds to these ligands, triggering the PI3K-AKT pathway, which bypasses the need for traditional TCR activation and triggers an immediate cytotoxic response. The intestinal epithelial cells (IECs) secrete specific cytokines (like TGF-β) and tissue repair factors, and provide the distinct cytokine signals. T-IELs are enable with barrier repair, rapid cytokine response and immune regulation (**B**). The induced conventional T-IELs are dependent of activation of T-IELs upon antigen encounter in the periphery, they traffic to the mucosal epithelium (**C**). Once established within the epithelial layer, the epithelial cells that provide the distinct cytokine signals (like IL-15 and TGF-β) necessary to upregulate T-IELs tissue-retention markers like CD103+ and CD69+, in which the conventional T-IELs become resident T-IELs, and are enable with effector functions, such as cytotoxic properties, cytokine response, proliferation and patrol. AI Gemini 3.1. Pro Advanced.

T-cell-derived IELs (T-IELs) are an abundant, specialized population of immune cells situated on the mucosal epithelium of MALT (lung, gut, and reproductive tract) [25,37,38]. They are broadly divided into two distinct lineages based on their developmental origin: natural and induced. Briefly, natural T-IELs (nIELs), such as TCRγδ+ and TCRαβ+ CD8αα+ T cells, are “tissue-resident” from early development. They migrate to the epithelium directly from the thymus without needing prior activation by a foreign antigen. They act as innate-like first responders. On the contrary, induced T-IELs (iIELs) are conventional, adaptive T cells, primarily TCRαβ^+^ CD4+ or CD8αβ^+^, that originate in peripheral lymphoid organs (Figure 2A), and are positioned at the critical intersection between the host and the external environment, primarily acting a first line of defense [1,2,3,16,20,24,25]. During an immune response to an infection (microbial/viral), circulating naive CD4^+^ or CD8^+^αβ T cells recognize the pathogen, become activated, and undergo clonal expansion. Following the clearance of the infection, a fraction of these activated effector T cells migrates into the mucosal epithelium. Once they cross into the epithelial layer, the local microenvironment—driven by cytokines like TGF-β and IL-15—induces them to permanently reside there as T-IELs. During this process, they downregulate tissue-exiting receptors (like S1PR1) and upregulate residency markers, most notably CD69+ and CD103+ (αE integrin, which binds to E-cadherin on epithelial cells) [37,38]. Once established within the epithelial layer, forming a long-term memory population restricted to a specific tissue without recirculating in the blood, these memory cells are structurally and functionally classified as epithelial TRM cells (CD69+/CD103+). How do these epithelial TRM cells collaborate with the mucosal immune system to face the constant exposure to a myriad of antigens (microbial and viral) in either of the tracts of MALT. After secondary reencounter, or reinfection, TRM populations play a key role in enhanced adaptive memory and protective immune response in infectious disease, or in the mucosal vaccination (oral/nasal) of particulate antigens [33,34,35,36,39,40,41] (Figure 2A).

### 2.1. Heterogeneity of T-IELs in MALT That Depends of

#### 2.1.1. -Infection Site

The highly specific microenvironment and physiological demands of the different mucosal tissues influence these properties [40,41]. For example, in the gut, T-IELs are predominantly conventional TCR γδ^+^ CD8^+^ T cells, with a smaller fraction of TCR γδ^+^ CD4^+^ T cells. Of relevance is that for the gut mucosal immunity, “unconventional” T cells expressing the CD8αα homodimer with a high frequency play a role by conferring innate-like, regulatory features [25,26,27,28,29,41] (Figure 2B,C). Another feature of their heterogeneous functionality is that through continuous crosstalk with intestinal epithelial cells (IECs), gut T-IELs secrete specific cytokines (like TGF-β) and tissue repair factors that promote epithelial restitution and maintain tight junction integrity, leading to barrier homeostasis [40,41,42] (Figure 2A). Does this heterogeneity of IELs across different mucosal sites (the lung, gut, and reproductive tract) influence mucosal vaccine design? For example, during infections, TCR γδ^+^ T-IELs are required for optimal protection against several intestinal pathogens [11,23,26,34,43], and express γδ T-cell receptors and the integrin αEβ7 [25,43]. A recent report shows that sepsis induced the migration of intestinal γδ Th17 to the lung, causing an increase in IL17A production and therefore exacerbating inflammatory response in acute lung injury syndrome [20]. Interestingly, in the lung, bronchial interstitium and intestine, the predominant form of T-IELs is CD8^+^ cytotoxic/suppressor T cells [1,25,44,45,46] (Figure 2A–C). The respiratory epithelium harbors a lower overall density of T-IELs compared to the intestines, which consist significantly of CD4+ and CD8+ tissue-resident memory T cells (TRM) that establish long-term residence following previous respiratory infections (e.g., influenza) [45,46,47,48,49]. TCR γδ^+^ T cells are also present but are functionally distinct from their intestinal counterparts. Indeed, for respiratory viruses, type I IFNs arrest the viral cycle, followed by the production of cytolytic molecules (i.e., granzyme and perforin). Furthermore, in the lower airways, which are highly populated and regulated by resident T-IELs, these cells actively maintain epithelial barrier integrity (e.g., by regulating claudin-1 expression) and thus dampen overactive inflammatory responses to harmless inhaled environmental antigens (Figure 2A).

#### 2.1.2. -Type of Pathogen

Gut T-IELs during *Cryptosporidium* infection possess a highly activated, tissue-resident memory phenotype (e.g., expressing CD69^+^ and CD103^+^), functioning as innate-like sentinels capable of mounting rapid, perforin/granzyme-mediated cytolytic attacks against infected or stressed epithelial cells without requiring classical clonal expansion [23,24,25,26,27,28] (Figure 2B,C).

T-IELs along with other types of lymphocytes and NK cells, experience significant depletion [48,49] against a member of the filovirus family which causes severe gastrointestinal dysfunction, often leading to intense diarrhea and life-threatening dehydration, T-IELs, along with other types of lymphocytes and NK cells, experience significant depletion [48,49]. The T-IEL cell population is relevant for promoting an antiviral state by upregulating interferon-responsive genes in the epithelium, enabling activated IEL mediators to protect surrounding cells against virus infections [50,51]; the cytotoxic and non-cytolytic antiviral activities of γδ^+^ T-IEL cells are critical in protecting the host against diverse viral pathogens [50]. 

Although some T-IELs (Ly6A+CCR9+CD4+) have a protective role against enteric adenovirus and reovirus infections, T-IELs role in resistance against viral infections remains elusive [50,51]. The regulated expression of the inducible costimulatory (ICOS) molecule is associated with CD43^+^ T-IELs; and that stimulation of T-IELs through ICOS enhances IFN-γ synthesis during infection [50]. In the exploration of human papillomavirus infections, higher cervical T-IEL numbers are associated with spontaneous regression of cervical intraepithelial neoplasia, a cervical cancer precursor lesion; T-IELs increased significantly in the patients whose neoplasia lesions regressed spontaneously regardless of human papillomavirus genotype [52].

In AIDS, the total number of CD3^+^ T-IELs was significantly reduced, and a relative expansion of the γδ^+^ T-IEL fraction occurred in advanced AIDS at the same time as the total T-IEL population was reduced; thus, γδ^+^ T-IELs are largely innate cells in first-line microbial defense [28,29,43]. It is known that during infections, a complex and dynamic relationship exists between intestinal epithelial cells (IECs) and intestinal lymphoid cells (T-IELs) where IEC sensing of microbes determines T-IEL distribution, migration, and energy utilization [13,16]. One viral infection exhibiting this dynamic is COVID-19, caused by SARS-CoV-2. During viral propagation, various components of intestinal immunity are affected, resulting in an abnormal or altered immune response. Although the role of T-IELs has not yet been fully defined, it is hypothesized that it induces IEC apoptosis [34], since intestinal biopsies from infected patients showed an increase in T-IELs along with reduced levels of key inflammatory proteins in circulation [53]. Another study confirmed the increase in CD8^+^ T-IELs activated upon infection of intestinal epithelial cells with SARS-CoV-2 as well as epithelial apoptosis [24,25,26]. Regarding beneficial microorganisms, although the role of commensal viruses in health and disease is understudied, it is known that these entities are essential for T-IEL homeostasis at the interface of mucosal epithelial tissues [28,29,43,49,50,51,54]. How to reach them? The most promising strategy is through mucosal vaccination, along with prime boost protocols, to ensure the induction of innate and cellular adaptive immunity.

In bacterial infections, the induction of immune response aimed at pathogen clearance is through priming respiratory T-IELs for the induction of pro-inflammatory response, represented by IFN-γ (*M. tuberculosis*) [44]. T-IELs in FRT achieve dual delicate physiological balance. On one hand, they provide essential mucosal defense against sexually transmitted infections (such as HIV, HPV, and *Neisseria gonorrhoeae*) via rapid cytolysis and the secretion of pro-inflammatory mediators. On the other hand, they concurrently exert immune tolerance, particularly during the secretory phase of the menstrual cycle [42]. In *Helicobacter pylori* (*H. pylori*) infection and coeliac disease (CD), it was observed that during antibiotic treatment, duodenal T-IELs were reduced [33,44], and it was discovered that there were significant changes in the T-IEL subpopulations [32,44]. Furthermore, in HP-associated gastritis and HP-associated lymphocytic gastritis, enhanced T-IEL cytotoxicity was observed along with enhanced granzyme B-associated cytotoxicity, thus contributing to an increase in epithelial apoptosis [38,39,40]. In addition, there are findings suggesting that, in patients with small intestinal bacterial overgrowth, γδ^+^ T-IELs might have a key role against intestinal bacterial infections [23,28,41]. Likewise, it has been shown that in oral infection by *Salmonella enterica* serovar *typhimurium*, which causes gastrointestinal diseases in humans, the expansion of intestinal T-IELs, particularly CD8^+^ γδ^+^ T-IELs, was promoted, which are cells that play important roles in the detection of pathogenic bacteria and the eradication of infected epithelial cells and, thus, provide protection against invading pathogens [13,34,43,44]. In another study with a murine model of infection with *Salmonella typhimurium*, it was observed that the migration of γδ^+^ T-IELs rapidly localized to and remained near epithelial cells in direct contact with *Salmonella typhimurium*, actions that limit bacteria invasion [25,37,38,39,43,44]. In this context of cell migration, it was also discovered that γδ^+^ T-IELs exhibit unique microbiota-dependent location and movement patterns in the epithelial compartment, and that the behavioral pattern quickly changes upon exposure to different enteric pathogens, resulting in increased intraepithelial cell scanning, expression of antimicrobial genes, and glycolysis. T-IELs are, therefore, highly mobile and display a structured migration pattern, suggesting an epithelial surveillance program [28,34,39,43,44]. A γδ^+^ T-IEL hyper-proliferative phenotype has also been identified, and this T cell population is capable of increased migratory behavior, leading to enhanced protection against bacterial infection [20,34,43,44,55] (Figure 2B,C).

## 3. Mucosal Vaccination and Prime Boost Strategies for the Induction of Innate and Adaptive Memory

**Figure 3:**
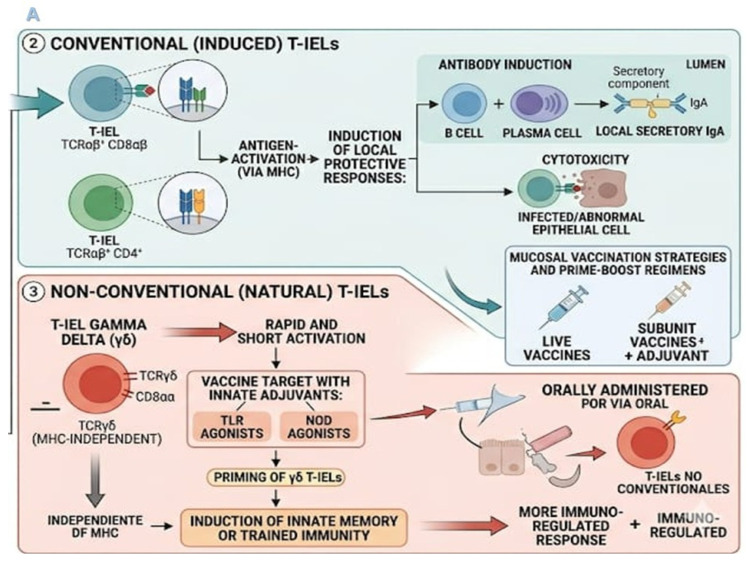

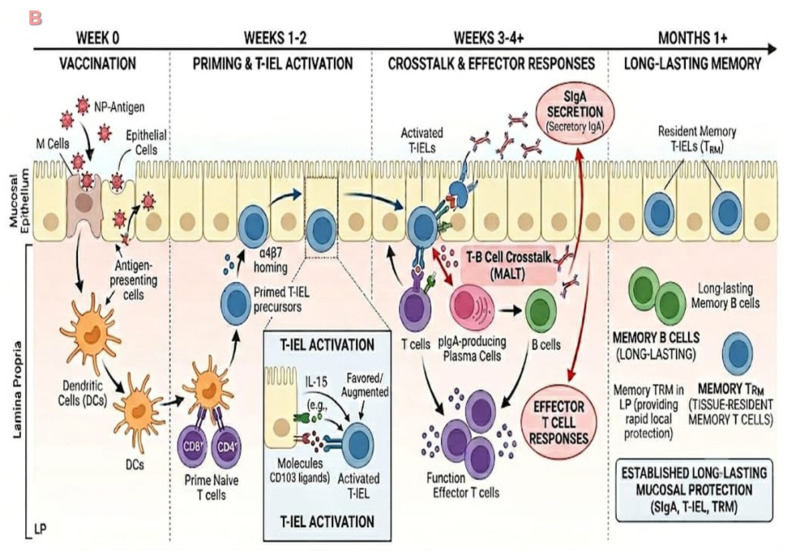
(**A**) Conventional T-IELs induce local protective responses of antibodies and cytotoxicity, and they are activated and induced by antigens. In terms of the mucosal vaccination and prime boost regimens to activate them, mostly live vaccines or subunit vaccines plus adjuvant are used, thus producing local humoral and cellular immune responses. Non-conventional T-IELs are natural are primed with a rapid and short activation, targeted through vaccines with innate adjuvants, such as TLR agonists, or NOD agonist. Gamma-delta T-IELs are endowed with the capacity to induce innate memory or trained immunity, and are independent of MHC. Alternatively, vaccines administered by oral route target T-IELs but with a more immune regulated response. Mucosal vaccination (intranasal/oral) allows researchers to target the inductive sites of the intestine or lung tissue in secondary lymphoid tissues (lymph nodes, mesenteric, and Peyer’s patches, PP) toward the activation of naïve CD4+ T cells and further expression of the gut-homing receptors (like α4β7) and mucosal retention markers (CD103+) to populate the effector sites (lamina propria of MALT). (**B**) Targeting and activation of T-IELs through mucosal vaccination. In the course of time (weeks or months), how is the generation, priming and activation of T-IELs after vaccination and the interaction with T-IELs and epithelial cells favored or their activation augmented, establishing further crosstalk with T and B cell responses for SIgA and effector T cell responses, especially long-lasting memory B cells responses and memory TRM in the lamina propria of MALT? Tissue-resident memory T cells (TRM) in MALT constitute a major subset of the T-intraepithelial cells that express mucosal tissue retention signals, and their activated state prompts a response under a second Ag encounter and a boost strategy. Induced conventional CD8+ cytotoxic cells are enabled with memory inducing properties, while the induced conventional CD4+ helper T cells coordinate local immune responses via cytokines and help B cells to produce mucosal IgA antibodies. These properties of both CD8+ and CD4+ T cells are favored upon second encounter or boosting, leading to tissue-resident memory (TRM) cells by expressing tissue-dependent α4β7 var/CD103+, IFN-γ, TNF-alpha, and IL-17. AI Gemini 3.1. Pro Advanced.

Mucosal vaccination and prime boost protocols are unique in the induction of long-lasting memory responses, in terms of antibodies and cellular immune responses, which are antigen-specific and MHC-dependent. The question is whether through these potential and effective strategies it is possible to induce innate memory immunity or “trained immunity” in which innate cells play a key role. Another type of innate cells, a special innate lymphocyte population are the gamma-delta T cells which are endowed with genes encoding innate receptors [56,57] (Figure 3A). These cells upon infection are able to recognize invaders at mucosal surfaces on MALT, activate, and trigger the expression of innate immune receptors (e.g., NKG2C and NKG2D), Toll-like receptors (TLR2, TLR3 and TLR 6), and nucleotide-binding oligomerization domain containing 2 (NOD) [58,59]. Can the presence of these innate receptors influence innate-like memory response of these T lymphocytes? Does trained immunity also apply to IELs? Can prime boost strategies enhance IEL function through the induction of trained immunity? If so, what are the potential molecular mechanisms? Moreover, metabolic rewiring to fulfill the energetic requirements, i.e., increased mitochondrial mass and spare respiratory capacity for memory T cells, both upon challenge and/or upon boosting, enhances proliferation and significantly produces effector molecules [60,61]. At this point, it is noteworthy to mention that the settings in which boosting is performed depend enormously on whether the boosting is homologous (live attenuated vaccines) or heterologous (glycoproteins antigens), as well as on the cellular components (virulence factors). These insights can aid in improving human therapeutic interventions in infectious diseases and in other types of non-infectious diseases. Thus, it has been reported that after subcutaneous vaccination with BCG vaccine in pre-weaned calves, the γδ T cells play a role in the induction of innate immunity. This was associated with increased chromatin accessibility of innate immune response-related genes, thereby increasing IL-6 and TNF-α cytokine production. Tissue-resident memory T cells (TRM), as mentioned before, are conventional intraepithelial lymphocytes induced by antigens that migrate to the epithelium, where they express markers of tissue residence. After reinfection, mucosal vaccination or boosting can influence the desired outcome of the local immune response, such as the induction of mucosal secretory IgA (SIgA) and the neutralization of systemic antibodies (IgG) against diverse pathogens [62,63,64,65] (Figure 3A,B).

### Targeting T-IELs Through Mucosal Vaccination Depends on How the Immune System Is Priming. To Perform This, the Type of Antigen, System Delivery and Route of Vaccination Play a Pivotal Role (Figure 3A)

- Type of antigen. Antigens are used to mimic natural infection as live vectors and induce the proliferative burst and efficient effector and memory B and T cells in mucosal tissues. In addition, live vectors actively infect host cells or actively cross mucosal barriers (mimicking a natural infection), and provide massive amounts of endogenous danger signals (PAMPs). This triggers an immediate and robust innate immune cascade that drives intense T cell clonal expansion. A recent report showed that by mining the CD4+ T cell repertoire, it is possible to screen TB protective antigens, with an augmented and superior performance to the BCG vaccine. These antigens are also recognized in humans exposed to *M. tuberculosis*.
- System delivery. As a part of the vaccine formulation, a drug delivery formulation specifically targets the M cells on the mucosal epithelium of GALT [66,67,68,69,70] (Figure 3B). These Ag vaccine formulations utilize distinct immunological mechanisms but essentially rely on the basic and fundamental aims, that is, to mimic utmost natural infections (microbial or viral infectious disease) [71] and to activate the entire immune system, innate and adaptive, resulting in an inflammatory, and effector and memory immune response [53,71,72]. In the vaccine formulation, adjuvants on adenoviral-vectored (i.e., COVID-19 or measles) [73,74,75,76,77,78,79] multiepitope vaccines can also be used. The mechanism relies on a particle aided by the adjuvants (such as aluminum, chitosan, CpG polymers, ISCOMS, recombinant bacteria, or inactivated toxins) [76,78,80,81] toward passive uptake by mucosal antigen-presenting cells (APCs), leading to the induction of a pro-inflammatory response (IL-6, IL-12, and TNF-α), and then the activation of the cellular immune response, in terms of cytokines (IFN-γ, IL-4, IL-15, IL-18) for the differentiation of naïve CD4+ T cells; subsets of Th1, Th2, Th3, and Th17, for the activation of CD8^+^ T cells; and iNKT, NK, and TRM/IELs, for the differentiation of plasmacytoid B cells and the production of antibodies (sIgA) [69,79,82] (Figure 3B).
- By other hand, mucosal vaccines depend heavily on the delivery platform such as viral vectors, bacterial vectors, and nanoparticles to activate and establish the TRM through mucosal vaccination [1,2,3,28,52,66,67,68,70,83]. Nanoparticles are used as live vectors to mimic natural infection and induce the proliferative burst and efficient effector and memory B and T cells in mucosal tissues. There is also a novel delivery system based on the yeast *Saccharomyces cerevisiae* (*S. cerevisiae*) for oral vaccine formulations. This system is safe and effective for delivering heterologous antigens (i.e., VP2 capsid protein) (PAMPS) as agonists of TLRs (PRRS) to induce systemic and mucosal immune responses, including antibody responses mainly of IgG and IgA [84]. This type of mucosal adjuvant candidate has been formulated as nano-carriers or nanoparticles based on chitosan or viral particles [11,12,80,81,84,85,86,87,88,89,90,91], a formulation that can be accessible and taken up by M cells, and transported to the inductive sites in the lung or GALT. Regarding dry powder vaccines, or drug formulations for immunization by the nasal route, there are several advantages to this delivery system: the dose, the cost, and, importantly, the priming and induction of systemic and mucosal immune responses [80,92]. Several reports in the literature have described the use of next-generation vaccine platforms as a delivery system, based on microbial organisms (i.e., *Salmonella*) as carriers of specific heterologous antigens (i.e., outer membrane vesicles) and molecule-like receptors (i.e., FcRN) for the induction of opsonizing antibodies and cellular immune response (CD4^+^ T cells) [93,94,95] (Figure 3B).
- Route of vaccination. Vaccines administered by the mucosal route are sampled M or DCs cells in the lumen of the mucosal epithelium of MALT (gut, lung and urogenital tract) uptake and transported through mucus and across specialized epithelia to reach tissue-specific immune cells, lymphoid structures and secondary inductive sites in the lymph nodes (axial, close to BALT and lung) and Peyer’s patches in GALT. This results in the induction of protective and long-lasting memory immune responses of antibodies (mostly secretory IgA) (SIgA), and TRM [1,3,25,36,53]. Due to the heterologous boosting at local sites (mucosal administration versus intramuscular), secondary B cell responses are higher and show cross-reactivity compared to those after intramuscular boosting [96,97,98,99,100,101,102,103]. Intramuscular DNA vaccine priming and intranasal boosting with live attenuated virus influenza-vectored vaccine (LAIV) 1 induced migration of the systemic central memory T cells (TCM) via CXCR3-dependent chemotaxis to the local sites/MALT (lung mucosal epithelium) for differentiation into TRM, and increased CD8^+^ TRM cells in the lung [102,103]. In addition, priming and boosting for *M. tuberculosis* infection produced induction adaptive immune responses of CD8^+^ T cells and CD4^+^ T cells, while γδ IL-17-producing cells enable immune protective immune responses [96,99,104]. Of note, a vaccine formulation consisting of a nano-emulsion (NE) and an RNA-based RIG-I agonist (IVT), a protein-based SARS-CoV-2 vaccine, with priming by the parenteral route (intramuscular), initiates a route that can be rerouted by IN immunization, driving the maturation of B and T cellular immune responses, to a protective and memory local response against Orto poxviruses (OPXVs) and against SARS-CoV-2 [82,100,105,106,107]. In either case, an increase in anti-S IgG immune status ratio (ISR) [79,82] and effective production of neutralizing antibodies were observed in a murine model [100]. Moreover, using a prime boost regimen against the chicken infectious anemia virus [99], consisting of DNA priming and boosting with a recombinant protein, elicited higher antibody titers and significant amounts of IL2, IL4, and IFN-γ [101]. Studies have shown that it is possible to induce airway luminal T cell responses accompanied by the induction of Th17-mediated IL-17 and IL-23, which has been shown to protect mice challenged with *M. tuberculosis* [105,106,107], by combining the parenteral route of immunization with priming based on attenuated microorganisms, followed by IN boosting with recombinant proteins [79,82,108,109] (Figure 3B).

## 4. Remarks

T-IELs as tissue-resident T cells perform constant immune surveillance to maintain and preserve epithelial integrity when any external or internal antigen (e.g., harmful pathogens or commensals) may compromise the mucosal barrier in MALT (gut, lung and urogenital tract).

- The challenges and limitations of targeting T-IELs through mucosal vaccination and prime boost strongly are dictated by the nature, the dose and the formulation of the vaccine, to avoid inducing excessive inflammation or autoimmunity. In fact, the dichotomous role of T-IELs is to maintain homeostasis and tolerance at the mucosal epithelium. Usually upon mucosal vaccination, vaccine antigens should be optimized to be delivered as a particulate formulation to reach the inductive sites, under the premise that the role of vaccination is to mimic to the utmost the microbial/viral infectious disease.
- How can these strategies really enhance proliferation, activation and memory responses? Upon oral/nasal vaccination of a particulate system (liposomes, viral particles, or nanoparticles), the innate immune cells, the APCs, M, epithelial cells and the arms of dendritic cells take the antigen up, transport it to the inductive sites and present it to T-IELs, which already are in a threshold activation state, ready to act.
- How they can react to this antigen presentation, and to repeated antigen vaccination, will depend strongly on the dosification of the stimulus and the route of immunization. For example, heterologous prime boost protocols, IM/IN, or IN/IN, have been shown to induce protection against challenges of *M. tuberculosis*, *Salmonella typhymurium*, and other viruses, mostly due to the activation and induction of γδT-IEL Th-17 cells.
- One of the hallmarks of the recent vaccine development is to take into account that vaccine candidates must mimic to the utmost the microbial/virus or the pathogens. Particulate formulations, based on liposomes or nanoparticles, are chosen to trigger and activate systemic and mucosal immunity; otherwise, they may reach the systemic compartments but not MALT.
- The development of vaccines and immunotherapies has been accelerated by the use of artificial intelligence, speeding up the design, modeling, and prediction of effectiveness in preclinical animal models. Furthermore, recent reports have shown that mining the CD4 repertoire systematically screened for protective Tb antigens in preclinical models, which are also recognized in humans exposed to *M. tuberculosis*, which showed higher and enhanced protection than the BCG vaccine alone. These results provide a novel platform of trivalent mRNA–lipid nanoparticle (RNA-LNP) vaccines for clinical development.

## Data Availability

Based on searches and data from the PubMed database without limitation to 2026.

## References

[1] Leceta J., Del Campo R., Jordan S., Klose C.S.N. (2022). Editorial: Immunoregulation at mucosal surfaces. Front. Immunol..

[2] Smith P., Blumberg R., MacDonald T., Society for Mucosal Immunology (2020). Principles of Mucosal Immunology.

[3] Allie S.R., Randall T.D. (2020). Resident Memory B Cells. Viral Immunol..

[4] Gozzi-Silva S.C., Teixeira F.M.E., Duarte A.J.D.S., Sato M.N., Oliveira L.M. (2021). Immunomodulatory Role of Nutrients: How Can Pulmonary Dysfunctions Improve?. Front. Nutr..

[5] Ansaldo E., Farley T.K., Belkaid Y. (2021). Control of Immunity by the Microbiota. Annu. Rev. Immunol..

[6] Ma H., Qiu Y., Yang H. (2021). Intestinal intraepithelial lymphocytes: Maintainers of intestinal immune tolerance and regulators of intestinal immunity. J. Leukoc. Biol..

[7] Han M., Rajput C., Ishikawa T., Jarman C.R., Lee J., Hershenson M.B. (2018). Small Animal Models of Respiratory Viral Infection Related to Asthma. Viruses.

[8] Marozzi M., Parnigoni A., Negri A., Viola M., Vigetti D., Passi A., Karousou E., Rizzi F. (2021). Inflammation, Extracellular Matrix Remodeling, and Proteostasis in Tumor Microenvironment. Int. J. Mol. Sci..

[9] Omahdi Z., Horikawa Y., Nagae M., Toyonaga K., Imamura A., Takato K., Teramoto T., Ishida H., Kakuta Y., Yamasaki S. (2020). Structural insight into the recognition of pathogen-derived phosphoglycolipids by C-type lectin receptor DCAR. J. Biol. Chem..

[10] Sadhu S., Kumar S., Mitra D.K., Joshi B. (2022). Activated TLR2/4-positive T cells boost cell exhaustion during lepromatous leprosy infection via PD-1 upregulation. Heliyon.

[11] Xie B., Wang M., Zhang X., Zhang Y., Qi H., Liu H., Wu Y., Wen X., Chen X., Han M. (2024). Gut-derived memory gammadelta T17 cells exacerbate sepsis-induced acute lung injury in mice. Nat. Commun..

[12] Kumar V. (2020). Pulmonary Innate Immune Response Determines the Outcome of Inflammation during Pneumonia and Sepsis-Associated Acute Lung Injury. Front. Immunol..

[13] Fischer M.A., Golovchenko N.B., Edelblum K.L. (2020). γδ T cell migration: Separating trafficking from surveillance behaviors at barrier surfaces. Immunol. Rev..

[14] Balaji S., Cholan P.K., Victor D.J. (2021). An emphasis of T-cell subsets as regulators of periodontal health and disease. J. Clin. Transl. Res..

[15] Mintz M.A., Cyster J.G. (2020). T follicular helper cells in germinal center B cell selection and lymphomagenesis. Immunol. Rev..

[16] Planer J.D., Morrisey E.E. (2023). After the Storm: Regeneration, Repair, and Reestablishment of Homeostasis Between the Alveolar Epithelium and Innate Immune System Following Viral Lung Injury. Annu. Rev. Pathol..

[17] Dizaji Asl K., Mazloumi Z., Majidi G., Kalarestaghi H., Sabetkam S., Rafat A. (2022). NK cell dysfunction is linked with disease severity in SARS-CoV-2 patients. Cell Biochem. Funct..

[18] Li Y., Handley S.A., Baldridge M.T. (2021). The dark side of the gut: Virome-host interactions in intestinal homeostasis and disease. J. Exp. Med..

[19] Ivanov I.I., Tuganbaev T., Skelly A.N., Honda K. (2022). T cell Responses to the Microbiota. Annu. Rev. Immunol..

[20] Yu Z., Chen J., Liu Y., Meng Q., Liu H., Yao Q., Song W., Ren X., Chen X. (2023). The role of potential probiotic strains *Lactobacillus reuteri* in various intestinal diseases: New roles for an old player. Front. Microbiol..

[21] Mikami Y., Kuroda E., Kimura S., Hayatsu M., Watanabe K., Tsuda H., Nakamura Y., Sagawa T., Ichinose T., Takano H. (2025). Bronchus-associated lymphoid tissue: A review of its development and function, including recent findings on the impact of environmental particulate exposure. Clin. Exp. Med..

[22] Han S. (2024). Unveiling an Important New Cell Type in the Lung: Microfold Cells. Am. J. Respir. Cell Mol. Biol..

[23] Johnson M.D., Witherden D.A., Havran W.L. (2020). The Role of Tissue-resident T Cells in Stress Surveillance and Tissue Maintenance. Cells.

[24] McArdle S., Seo G.Y., Kronenberg M., Mikulski Z. (2023). Intravital Imaging of Intestinal Intraepithelial Lymphocytes. Bio-Protoc..

[25] Vandereyken M., James O.J., Swamy M. (2020). Mechanisms of activation of innate-like intraepithelial T lymphocytes. Mucosal Immunol..

[26] Nazmi A., McClanahan K.G., Olivares-Villagomez D. (2021). Unconventional Intestinal Intraepithelial Lymphocytes in Health and Disease. Crit. Rev. Immunol..

[27] Nie J., Carpenter A.C., Chopp L.B., Chen T., Balmaceno-Criss M., Ciucci T., Xiao Q., Kelly M.C., McGavern D.B., Belkaid Y. (2022). The transcription factor LRF promotes integrin β7 expression by and gut homing of CD8αα^+^ intraepithelial lymphocyte precursors. Nat. Immunol..

[28] Contreras A.V., Wiest D.L. (2023). Development of γδ T Cells: Soldiers on the Front Lines of Immune Battles. Methods Mol. Biol..

[29] Lai A.Y., Patel A., Brewer F., Evans K., Johannes K., González L.E., Yoo K.J., Fromm G., Wilson K., Schreiber T.H. (2022). Cutting Edge: Bispecific γδ T Cell Engager Containing Heterodimeric BTN2A1 and BTN3A1 Promotes Targeted Activation of Vγ9Vδ2^+^ T Cells in the Presence of Costimulation by CD28 or NKG2D. J. Immunol..

[30] Sun S., Li E., Zhao G., Tang J., Zuo Q., Cai L., Xu C., Sui C., Ou Y., Liu C. (2023). Respiratory mucosal vaccination of peptide-poloxamine-DNA nanoparticles provides complete protection against lethal SARS-CoV-2 challenge. Biomaterials.

[31] Lei H., Alu A., Yang J., Ren W., He C., Lan T., He X., Yang L., Li J., Wang Z. (2022). Intranasal administration of a recombinant RBD vaccine induces long-term immunity against Omicron-included SARS-CoV-2 variants. Signal Transduct. Target. Ther..

[32] Dijkgraaf F.E., Kok L., Schumacher T.N.M. (2021). Formation of Tissue-Resident CD8^+^ T-Cell Memory. Cold Spring Harb. Perspect. Biol..

[33] Wijeyesinghe S., Beura L.K., Pierson M.J., Stolley J.M., Adam O.A., Ruscher R., Steinert E.M., Rosato P.C., Vezys V., Masopust D. (2021). Expansible residence decentralizes immune homeostasis. Nature.

[34] Crowl J.T., Heeg M., Ferry A., Milner J.J., Omilusik K.D., Toma C., He Z., Chang J.T., Goldrath A.W. (2022). Tissue-resident memory CD8^+^ T cells possess unique transcriptional, epigenetic, and functional adaptations to different tissue environments. Nat. Immunol..

[35] Palgen J.L., Feraoun Y., Dzangué-Tchoupou G., Joly C., Martinon F., Le Grand R., Beignon A.S. (2021). Optimize Prime/Boost Vaccine Strategies: Trained Immunity as a New Player in the Game. Front. Immunol..

[36] Kuraoka M., Yeh C.H.-Y., Bajic G., Kotaki R., Song S.H., Windsor I., Harrsion S.C., Kelsoe G. (2022). Recall of B cell memory depends on relative locations of prime and boost immunization. Sci. Immunol..

[37] Chowdhury R., Valainis J.R., Dubey M., von Boehmer L., Sola E., Wilhelmy J., Guo J., Kask O., Ohanyan M., Sun M. (2023). NK-like CD8^+^ gammadelta T cells are expanded in persistent *Mycobacterium tuberculosis* infection. Sci. Immunol..

[38] Zhou D.-Y., Bao C.-F., Zhou G. (2025). Intraepithelial lymphocytes in human oral diseases. Front. Immunol..

[39] Li G.Q., Xia J., Zeng W., Luo W., Liu L., Zeng X., Cao D. (2023). The intestinal γd T cells: Functions in the gut and in the distant organs. Front. Immunol..

[40] Dickson K.B., Stadnyk A.W., Zhou J., Lehmann C. (2025). Mucosal immunity: Lessons from the lower respiratory and small intestinal epithelia. Biomedicines.

[41] Bordoni D., Fazio A. (2025). Crosstalk between T cell gene regulation and intestinal epithelial cells: Insights into mucosal immunity. Advances in Genetics.

[42] Visnyaiová K., Varga I., Feitscherova C., Pavlikova L., Zahumensky J., Mikušova R. (2024). Morphology of the immune cells in the wall of the human uterine tube and their possible impact on reproduction—Uterine tube as a possible immune privileged organ. Front. Cell Dev. Biol..

[43] Lockhart A., Mucida D., Bilate A.M. (2024). Intraepithelial Lymphocytes of the Intestine. Annu. Rev. Immunol..

[44] Yilmaz F., Atay K., Çirkin G., Sanmak E. (2025). The impact of gastric *Helicobacter pylori* infection on duodenal mucosa: New evidence on the alteration of intraepithelial lymphocytes. Am. J. Clin. Pathol..

[45] Livanos A.E., Jha D., Cossarini F., Gonzalez-Reiche A.S., Tokuyama M., Aydillo T., Parigi T.L., Ladinsky M.S., Ramo I., Dunleavy K. (2021). Intestinal host response to SARS-CoV-2 infection and COVID-19 outcomes in patients with gastrointestinal symptoms. Gastroenterology.

[46] Lehmann M., Allers K., Heldt C., Meinhardt J., Schmidt F., Rodriguez-Sillke Y., Kunkel D., Schumann M., Chotima Böttcher Stahl-Hennig C.H., Elezkurtag S. (2021). Human small intestinal infection by SARS-CoV-2 is characterized by a mucosal infiltration with activated CD8^+^ T cells. Mucosal Immunol..

[47] Flores E.Y., Hume A.J., Olejnik J., Mithal A., D’Amico A., Yang M., Bawa P., Wang F., O’Connell A.K., Tseng A. (2025). Filovirus infection disrupts epithelial barrier function and ion transport in human iPSC-derived gut organoids. PLoS Pathog..

[48] Perez-Valencia L.J., Vannella K.M., Ramos-Benitez M.J., Sun J., Abu-Asab M., Dorward D.W., Awad K.S., Platt A., Jacobson E., Kindrachuk J. (2023). Ebola virus shed glycoprotein is toxic to human T, B, and natural killer lymphocytes. iScience.

[49] Von Massow G., Oh S., Lam A., Gustafsson K. (2021). Gamma Delta T Cells and Their Involvement in COVID-19 Virus Infections. Front. Immunol..

[50] Parsa R., London M., Rezende de Castro T.B., Reis B., Buissant des Amorie J., Smith J.G., Mucida D. (2022). Newly recruited intraepithelial Ly6A^+^CCR9^+^CD4^+^ T cells protect against enteric viral infection. Immunity.

[51] Eleftheriotis G., Tsounis E.P., Aggeletopoulou I., Dousdampanis P., Triantos C., Mouzaki A., Marangos M., Assimakopoulos S.F. (2023). Alterations in gut immunological barrier in SARS-CoV-2 infection and their prognostic potential. Front. Immunol..

[52] Al-Nemrawi N.K., Darweesh R.S., Al-Shriem L.A., Al-Qawasmi F.S., Emran S.O., Khafajah A.S., Abu-Dalo M.A. (2022). Polymeric Nanoparticles for Inhaled Vaccines. Polymers.

[53] Matsumoto R., Ogata K., Takahashi D., Kinashi Y., Yamada T., Morita R., Tanaka K., Hattori K., Endo M., Fujimura Y. (2024). AP-1B regulates interactions of epithelial cells and intraepithelial lymphocytes in the intestine. Cell. Mol. Life Sci..

[54] Rani S.B.E., Diaz F.E., Maina T.W., Corbett R.J., Tuggle C.H.K., McGill J.L. (2024). Evidence of innate training in bovine γδ T cells following subcutaneous BCG administration. Front. Immunol..

[55] Jia L., Wu G., Alonso S., Zhao C., Lemenze A., Lam Y.Y., Zhao L., Edelblum K.L. (2022). A transmissible γδ intraepithelial lymphocyte hyperproliferative phenotype is associated with the intestinal microbiota and confers protection against acute infection. Mucosal Immunol..

[56] Suen T.K., Al B., Scarpa A., Dorhoi A., Netea M.G., Palcek K. (2025). A dual nature of γδ T cell immune memory responses. eLife.

[57] Netea M.G., Domínguez- Andreés J., Barreiro L.B., Chavakis T., Divangahi M., Fuchs E., Joosten L.A.B., van der Meer J.W.M., Mhlanga M.M., Mulder W.J.M. (2020). Defining trained immunity and its role in health and disease. Nat. Rev. Immunol..

[58] Guo J., Chowdhury R.R., Mallajosyula V., Xie J., Dubey M., Liu Y., Li J., Wei Y., Palanski B.A., Wang C. (2024). γδ T cell antigen receptor polyspecificity enables T cell responses to a broad range of immune challenges. Proc. Natl. Acad. Sci. USA.

[59] Serrano R., Lettau M., Zarobkiewicz M., Wesch D., Peters C., Kabelitz D. (2022). Stimulatory and inhibitory activity of STING ligands on tumor- reactive human gamma/delta T cells. Oncoimmunology.

[60] Bevington S.L., Fiancette R., Gajdasik D.W., Keane P., Soley J.K., Willis C.M., Coleman D.J.L., Withers D.R., Cockerill P.N. (2021). Stable Epigenetic Programming of Effector and Central Memory CD4 T Cells Occurs Within 7 Days of Antigen Exposure *In Vivo*. Front. Immunol..

[61] Mittelstaedt N.N., Becker A.L., de Freitas D.N., Zanin R.F., Stein R.T., Duarte de Souza A.P. (2021). DNA Methylation and Immune Memory Response. Cells.

[62] Hassert M., Harty J.T. (2022). Tissue resident memory T cells- A new benchmark for the induction of vaccine-induced mucosal immunity. Front. Immunol..

[63] Zhou Y., Qu J., Sun X., Yue Z., Liu Y., Zhao K., Yang F., Feng J., Pan X., Jin Y. (2023). Delivery of spike-RBD by bacterial type three secretion system for SARS-CoV-2 vaccine development. Front. Immunol..

[64] Lublin A., Katz C., Gruzdev N., Yadid I., Bloch I., Farnoushi Y., Simanov L., Berkowitz A., Elyahu D., Pitcovski J. (2022). Protection against avian coronavirus conferred by oral vaccination with live bacteria secreting LTB-fused viral proteins. Vaccine.

[65] Peng Z., Cao D.Y., Wu H.Y., Saito S. (2020). Immunization with a Bacterial Lipoprotein Establishes an Immuno-Protective Response with Upregulation of Effector CD4+ T Cells and Neutrophils Against Methicillin-Resistant *Staphylococcus aureus* Infection. Pathogens.

[66] McCright C.J., Maisel K. (2020). Engineering drug delivery systems to overcome mucosal barriers for immunotherapy and vaccination. Tissue Barriers.

[67] Saggese A., Baccigalupi L., Donadio G., Ricca E., Isticato R. (2023). The Bacterial Spore as a Mucosal Vaccine Delivery System. Int. J. Mol. Sci..

[68] Tang W., Zhang Y., Zhu G. (2022). Pulmonary delivery of mucosal nanovaccines. Nanoscale.

[69] Vidal S.J., Lasrado N., Tostanoski L.H., Chaudari J., Mbiwan E.R., Neka G.D., Strutton E.A., Espinoza P.A.A., Sellers D., Barrett J. (2025). Mining the CD4 antigen repertoire for next generation tuberculosis vaccines. Cell.

[70] Freire H.H., Roe E.F., Collier J.H. (2023). Expanding opportunities to engineer mucosal vaccination with biomaterials. Biomater. Sci..

[71] Sargazi S., Arshad R., Ghamari R., Rahdar A., Bakhshi A., Karkan S.F., Ajalli N., Bilal M., Díez-Pascual A.M. (2022). SiRNA-based nanotherapeutics as emerging modalities for immune-mediated diseases: A preliminary review. Cell Biol. Int..

[72] Hartwell B.L., Melo M.B., Xiao P., Lemnios A.A., Li N., Chang J.Y.H., Yu J., Gebre M.S., Chang A., Maiorino L. (2022). Intranasal vaccination with lipid-conjugated immunogens promotes antigen transmucosal uptake to drive mucosal and systemic immunity. Sci. Transl. Med..

[73] Afkhami S., Kang A., Jeyanathan V., Xing Z., Jeyanathan M. (2023). Adenoviral-vectored next-generation respiratory mucosal vaccines against COVID-19. Curr. Opin. Virol..

[74] Guan X., Verma A.K., Liu Q., Palacios M., Oddle A.E., Perlman S., Du L. (2025). Glycosylated Receptor-Binding-Domain-Targeting Mucosal Vaccines Protect Against SARS-CoV-2 Omicron and MERS-CoV. Vaccines.

[75] Lei H., Alu A., Yang J., He C., Shi J., Hong W., Peng D., Zhang Y., Liu J., Qin F. (2025). Intranasal Inoculation of Cationic Crosslinked Carbon Dots-Adjuvanted Respiratory Syncytial Virus F Subunit Vaccine Elicits Mucosal and Systemic Humoral and Cellular Immunity. MedComm.

[76] Zhao Y., Liu J., Peng C.H., Guo S.H., Wang B., Longping C., Wang Y., Tang H., Liu L., Pan Q. (2025). Cross-protection against homo and heterologous influenza viruses via intranasal administration of an HA chimeric multiepitope nanoparticle vaccine. J. Nanobiotechnol..

[77] Peng C., Tang F., Wang J., Cheng P., Wang L., Gong W. (2023). Immunoinformatic-Based Multi-Epitope Vaccine Design for Co-Infection of *Mycobacterium tuberculosis* and SARS-CoV-2. J. Pers. Med..

[78] Nakahashi O.R., Mori H., Yuki Y., Machita T., Kataka Y., Umemoto S.H., Uchida Y., Yamanoue T., Sawada S.H.-I., Ishige K. (2025). Cationic nanogel-based nasal therapeutic HPV vaccine prevents the development of cervical cancer. Sci. Transl. Med..

[79] Radaelli A., Zanotto C., Brambilla C., Adami T., Paolini F., Venuti A., Manuka A., Mehmeti I., De Giuli Morghen C. (2024). Different immunogens and prime-boost vaccination strategies affect the efficacy of recombinant candidate vaccines against pathogenic orthopoxviruses. Virol. J..

[80] Kazemi M., Madani R., Aghamaali M.R., Emami T., Golchinfar F., Heshmati L. (2022). Preparation and Characterization of Nanoliposome Containing Isolated VP1 Protein of Foot and Mouth Disease Virus as a Model of Vaccine. Arch. Razi Inst..

[81] Harrell J.E., Kurtz J.R., Bauer D.L., Prior J.T., Gellings P.S., Morici L.A., McLachlan J.B. (2021). An Outer Membrane Vesicle-Adjuvanted Oral Vaccine Protects Against Lethal, Oral *Salmonella* Infection. Pathogens.

[82] Jeyanathan M., Vaseghi-Shanjani M., Afkhami S., Grondin J.A., Kang A., D’Agostino M.R., Yao Y., Jain S., Zganiacz A., Kroezen Z. (2022). Parenteral BCG vaccine induces lung-resident memory macrophages and trained immunity via the gut-lung axis. Nat. Immunol..

[83] Xu J., Dai W., Wang Z., Chen B., Li Z., Fan X. (2011). Intranasal vaccination with chitosan-DNA nanoparticles expressing pneumococcal surface antigen a protects mice against nasopharyngeal colonization by Streptococcus pneumoniae. Clin. Vaccine Immunol..

[84] Cao P., Han F.Y., Grøndahl L., Xu Z.P., Li L. (2020). Enhanced Oral Vaccine Efficacy of Polysaccharide-Coated Calcium Phosphate Nanoparticles. ACS Omega.

[85] Wang N., Wei C., Zhang Z., Liu T., Wang T. (2020). Aluminum Nanoparticles Acting as a Pulmonary Vaccine Adjuvant-Delivery System (VADS) Able to Safely Elicit Robust Systemic and Mucosal Immunity. J. Inorg. Organomet. Polym. Mater..

[86] J L.A.A., Pa P., Seng C.Y., Rhee J.H., Lee S.E. (2025). Protein nanocages: A new frontier in mucosal vaccine delivery and immune activation. Hum. Vaccine Immunother..

[87] Cazzola M., Ora J., Calzetta L., Rogliani P., Matera M.G. (2022). The future of inhalation therapy in chronic obstructive pulmonary disease. Curr. Res. Pharmacol. Drug Discov..

[88] Feng F., Wen Z., Chen J., Yuan Y., Wang C., Sun C. (2022). Strategies to Develop a Mucosa-Targeting Vaccine against Emerging Infectious Diseases. Viruses.

[89] Parriott J.E., Stewart J.P., Smith D.D., Curran S.M., Bauer C.D., Wyatt T.A., Phillips J.A., Lyden E., Thiele G.M., Vetro J.A. (2022). Surface Modification of Biodegradable Microparticles with the Novel Host-Derived Immunostimulant CPDI-02 Significantly Increases Short-Term and Long-Term Mucosal and Systemic Antibodies against Encapsulated Protein Antigen in Young Naïve Mice after Respiratory Immunization. Pharmaceutics.

[90] Dałek P., Drabik D., Wołczańska H., Foryś A., Jagas M., Jędruchniewicz N., Przybyło M., Witkiewicz W., Langner M. (2022). Bioavailability by design—Vitamin D_3_ liposomal delivery vehicles. Nanomedicine.

[91] Zhang C., Zhang P.Q., Guo S., Zhao Z., Wang G.X., Zhu B. (2022). Dual-Targeting Polymer Nanoparticles Efficiently Deliver DNA Vaccine and Induce Robust Prophylactic Immunity against Spring Viremia of Carp Virus Infection. Microbiol. Spectr..

[92] Li H., Hua D., Qu Q., Cao H., Feng Z., Liu N., Huang J., Zhang L. (2023). Oral Immunization with Recombinant *Saccharomyces cerevisiae* Expressing Viral Capsid Protein 2 of Infectious Bursal Disease Virus Induces Unique Specific Antibodies and Protective Immunity. Vaccines.

[93] Jia Z., Ma C., Yang X., Pan X., Li G., Ma D. (2021). Oral Immunization of Recombinant *Lactococcus lactis* and *Enterococcus faecalis* Expressing Dendritic Cell Targeting Peptide and Hexon Protein of Fowl Adenovirus 4 Induces Protective Immunity Against Homologous Infection. Front. Vet. Sci..

[94] Kong D., Pan H., Wu H., Chen J. (2025). Engaging Broader Stakeholders to Accelerate Group a *Streptococcus* Vaccine Development. Vaccines.

[95] Coria L.M., Pueblas Castro C., Bruno L., Nakaya H.I., Pasquevich K.A., Cassataro J. (2025). Discovery of protease inhibitors from bacteria as novel adjuvants for oral vaccine formulations. Front. Immunol..

[96] Laher F., Bekker L.G., Garrett N., Lazarus E.M., Gray G.E. (2020). Review of preventative HIV vaccine clinical trials in South Africa. Arch. Virol..

[97] Liu Z., Lu L., Jiang S. (2023). Application of “B+1” heterologous boosting strategy for preventing infection of SARS-CoV-2 variants with resistance to broad-spectrum coronavirus vaccines. Emerg. Microbes Infect..

[98] Taus E., Hofmann C., Ibarrondo F.J., Gong L.S., Hausner M.A., Fulcher J.A., Krogstad P., Kitchen S.G., Ferbas K.G., Tobin N.H. (2023). Persistent memory despite rapid contraction of circulating T cell responses to SARS-CoV-2 mRNA vaccination. Front. Immunol..

[99] Launay O., Thill P. (2022). Heterologous prime boost COVID 19 vaccination. Infect. Dis. Now.

[100] Xu H., Yue M., Zhou R., Wang P., Wong M.Y., Wang J., Huang H., Chen B., Mo Y., Tam R.C. (2024). A Prime-Boost Vaccination Approach Induces Lung Resident Memory CD8^+^ T Cells Derived from Central Memory T Cells That Prevent Tumor Lung Metastasis. Cancer Res..

[101] Sharifi Aliabadi L., Karami M., Barkhordar M., Hashemi Nazari S.S., Kavousi A., Ahmadvand M., Vaezi M. (2023). Homologous versus Heterologous prime-boost COVID-19 Vaccination in autologous hematopoietic stem cell transplantation recipients: A blinded randomized controlled trial. Front. Immunol..

[102] Chiu N.C., Chi H., Tu Y.K., Huang Y.N., Tai Y.L., Weng S.L., Chang L., Huang D.T., Huang F.Y., Lin C.Y. (2021). To mix or not to mix? A rapid systematic review of heterologous prime-boost covid-19 vaccination. Expert Rev. Vaccines.

[103] Cottrell C.A., Hu X., Lee J.H., Skog P., Luo S., Flynn C.T., McKenney K.R., Hurtado J., Kalyuzhniy O., Liguori A. (2024). Heterologous prime-boost vaccination drives early maturation of HIV broadly neutralizing antibody precursors in humanized mice. Sci. Transl. Med..

[104] Liu L., Yin M., Li Y., Su H., Fang L., Sun X., Chang S., Zhao P., Wang Y. (2022). DNA Prime and Recombinant Protein Boost Vaccination Confers Chickens with Enhanced Protection against Chicken Infectious Anemia Virus. Viruses.

[105] Arroyo-Díaz N.M., Bachus H., Papillion A., Randall T.D., Akther J., Rosenberg A.F., León B., Ballesteros-Tato A. (2023). Interferon-gamma production by Tfh cells is required for CXCR3^+^ pre-memory B cell differentiation and subsequent lung-resident memory B cell responses. Immunity.

[106] Li F., Dang W., Du Y., Xu X., He P., Zhou Y., Zhu B. (2024). Tuberculosis Vaccines and T cell Immune Memory. Vaccines.

[107] Kaveh D.A., Garcia-Pelayo M.C., Bull N.C., Sanchez-Cordon P.J., Spiropoulos J., Hogarth P.J. (2020). Airway delivery of both a BCG prime and adenoviral boost drives CD4 and CD8 T cells into the lung tissue parenchyma. Sci. Rep..

[108] Kirk N.M., Huang Q., Vrba S., Rahman M., Block A.M., Murphy H., White D.W., Namugenyi S.B., Ly H., Tischler A.D. (2023). Recombinant Pichinde viral vector expressing tuberculosis antigens elicits strong T cell responses and protection in mice. Front. Immunol..

[109] Niu L., Li H., Guo J., Jia Z., Chang Q., Liu N., Zhang S., Ge J. (2026). Gut commensal Lactobacillus strain induces a balanced trained immunity phenotype to enhance vaccine efficacy through JAK-STAT-SOCS pathway. Probiot. Antimicrob. Proteins.

